# *N*′-(4-Chloro­benzyl­idene)furan-2-carbohydrazide monohydrate

**DOI:** 10.1107/S1600536810005532

**Published:** 2010-02-13

**Authors:** Jin-He Jiang

**Affiliations:** a Microscale Science Institute, Department of Chemistry and Chemical Engineering, Weifang University, Weifang 261061, People’s Republic of China

## Abstract

In the title compound, C_12_H_9_ClN_2_O_2_·H_2_O, the dihedral angle between the aromatic rings is 13.9 (2)° and an intra­molecular N—H⋯O hydrogen bond occurs. In the crystal structure, the components are linked by N—H⋯O, O—H⋯O and C—H⋯O hydrogen bonds.

## Related literature

For background to Schiff bases, see: Cimerman *et al.* (1997 ▶). For a related structure, see: Girgis (2006 ▶).
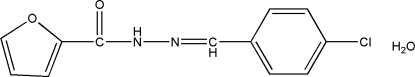


## Experimental

### 

#### Crystal data

C_12_H_9_ClN_2_O_2_·H_2_O
*M* *_r_* = 266.68
Monoclinic, 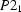

*a* = 4.5480 (9) Å
*b* = 12.423 (3) Å
*c* = 10.971 (2) Å
β = 91.90 (3)°
*V* = 619.5 (2) Å^3^
*Z* = 2
Mo *K*α radiation
μ = 0.31 mm^−1^
*T* = 293 K
0.25 × 0.20 × 0.18 mm

#### Data collection

Bruker SMART CCD diffractometer
5979 measured reflections
2799 independent reflections
1708 reflections with *I* > 2σ(*I*)
*R*_int_ = 0.042

#### Refinement

*R*[*F* ^2^ > 2σ(*F* ^2^)] = 0.045
*wR*(*F* ^2^) = 0.145
*S* = 1.04
2799 reflections
171 parameters
1 restraint
H atoms treated by a mixture of independent and constrained refinement
Δρ_max_ = 0.23 e Å^−3^
Δρ_min_ = −0.34 e Å^−3^
Absolute structure: Flack (1983 ▶), 1319 Friedel pairs
Flack parameter: 0.01 (11)

### 

Data collection: *SMART* (Bruker 1997 ▶); cell refinement: *SAINT* (Bruker 1997 ▶); data reduction: *SAINT*; program(s) used to solve structure: *SHELXS97* (Sheldrick, 2008 ▶); program(s) used to refine structure: *SHELXL97* (Sheldrick, 2008 ▶); molecular graphics: *SHELXTL* (Sheldrick, 2008 ▶); software used to prepare material for publication: *SHELXTL*.

## Supplementary Material

Crystal structure: contains datablocks global, I. DOI: 10.1107/S1600536810005532/hb5316sup1.cif

Structure factors: contains datablocks I. DOI: 10.1107/S1600536810005532/hb5316Isup2.hkl

Additional supplementary materials:  crystallographic information; 3D view; checkCIF report

## Figures and Tables

**Table 1:** Hydrogen-bond geometry (Å, °)

*D*—H⋯*A*	*D*—H	H⋯*A*	*D*⋯*A*	*D*—H⋯*A*
O1*W*—H1*W*⋯O1^i^	0.97 (5)	1.90 (5)	2.865 (4)	174 (5)
O1*W*—H2*W*⋯O1^ii^	0.92 (6)	1.97 (6)	2.873 (4)	168 (5)
N1—H1*A*⋯O2	0.86	2.36	2.715 (4)	106
N1—H1*A*⋯O1*W*	0.86	2.05	2.886 (4)	165
C8—H8*A*⋯O1*W*	0.93	2.49	3.290 (5)	144

Symmetry codes: (i) 
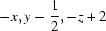
; (ii) 
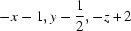
.

**Fig. 1.**
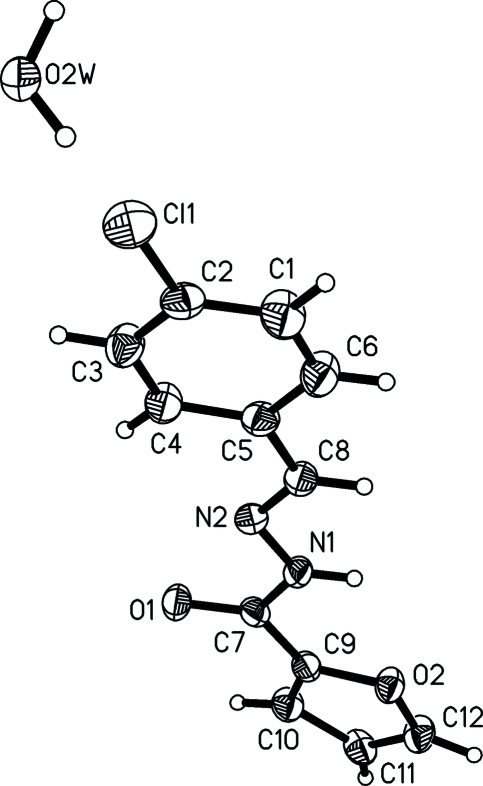
The structure of (I) showing 30% probability displacement ellipsoids.

## supplementary crystallographic information

### Comment

Schiff bases have received considerable attention in the literature. They are attractive from several points of view, such as the possibility of analytical application (Cimerman *et al.*, 1997). As part of our search for new schiff base compounds we synthesized the title compound (I), and describe its structure here.

The molcular structure of (I) is shown in Fig. 1. The C7—N1 bond length of 1.342 (4)Å is longer than the C—N double bond [1.281 (2) Å] reported (Girgis, 2006). In the crystal structure, molecules are linked by intermolecular N—H···O hydrogen bonds.

### Experimental

A mixture of furan-2-carbohydrazide (0.1 mol), and 4-chlorobenzaldehyde (0.1 mol) was stirred in refluxing ethanol (20 mL) for 4 h to afford the title compound (0.087 mol, yield 87%).  Colourless blocks of (I) were obtained by recrystallization from ethanol at room temperature.

### Refinement

H atoms were fixed geometrically and allowed to ride on their attached atoms, with C—H distances = 0.93-0.97 Å; N—H = 0.86Å and with *U*_iso_(H) = 1.2*U*_eq_(C,*N*) or 1.5*U*_eq_(C_methyl_).

### Crystal data

**Table:**

C_12_H_9_ClN_2_O_2_·H_2_O	*F*(000) = 276
*M**_r_* = 266.68	*D*_x_ = 1.430 Mg m^−^^3^
Monoclinic, *P*2_1_	Mo *K*α radiation, λ = 0.71073 Å
Hall symbol: P 2yb	Cell parameters from 2022 reflections
*a* = 4.5480 (9) Å	θ = 3.3–27.3°
*b* = 12.423 (3) Å	µ = 0.31 mm^−^^1^
*c* = 10.971 (2) Å	*T* = 293 K
β = 91.90 (3)°	Block, colourless
*V* = 619.5 (2) Å^3^	0.25 × 0.20 × 0.18 mm
*Z* = 2	

### Data collection

**Table:**

Bruker SMART CCD diffractometer	1708 reflections with *I* > 2σ(*I*)
Radiation source: fine-focus sealed tube	*R*_int_ = 0.042
graphite	θ_max_ = 27.5°, θ_min_ = 3.3°
ω scans	*h* = −5→5
5979 measured reflections	*k* = −16→16
2799 independent reflections	*l* = −14→14

### Refinement

**Table:**

Refinement on *F*^2^	Secondary atom site location: difference Fourier map
Least-squares matrix: full	Hydrogen site location: inferred from neighbouring sites
*R*[*F*^2^ > 2σ(*F*^2^)] = 0.045	H atoms treated by a mixture of independent and constrained refinement
*wR*(*F*^2^) = 0.145	*w* = 1/[σ^2^(*F*_o_^2^) + (0.0702*P*)^2^] where *P* = (*F*_o_^2^ + 2*F*_c_^2^)/3
*S* = 1.04	(Δ/σ)_max_ < 0.001
2799 reflections	Δρ_max_ = 0.23 e Å^−^^3^
171 parameters	Δρ_min_ = −0.33 e Å^−^^3^
1 restraint	Absolute structure: Flack (1983), 1319 Friedel pairs
Primary atom site location: structure-invariant direct methods	Flack parameter: 0.01 (11)

### Special details

**Table:**

Geometry. All esds (except the esd in the dihedral angle between two l.s. planes) are estimated using the full covariance matrix. The cell esds are taken into account individually in the estimation of esds in distances, angles and torsion angles; correlations between esds in cell parameters are only used when they are defined by crystal symmetry. An approximate (isotropic) treatment of cell esds is used for estimating esds involving l.s. planes.
Refinement. Refinement of *F*^2^ against ALL reflections. The weighted *R*-factor wR and goodness of fit *S* are based on *F*^2^, conventional *R*-factors *R* are based on *F*, with *F* set to zero for negative *F*^2^. The threshold expression of *F*^2^ > σ(*F*^2^) is used only for calculating *R*-factors(gt) etc. and is not relevant to the choice of reflections for refinement. *R*-factors based on *F*^2^ are statistically about twice as large as those based on *F*, and *R*- factors based on ALL data will be even larger.

### Fractional atomic coordinates and isotropic or equivalent isotropic displacement parameters (Å^2^)

**Table:**

	*x*	*y*	*z*	*U*_iso_*/*U*_eq_	
Cl1	0.8615 (3)	0.97667 (10)	0.39621 (9)	0.0713 (4)	
O1	−0.3072 (6)	0.94704 (18)	1.0474 (2)	0.0525 (6)	
O2	−0.5944 (6)	0.68010 (17)	1.0623 (2)	0.0457 (6)	
N1	−0.1934 (6)	0.7961 (2)	0.9399 (2)	0.0412 (6)	
H1A	−0.2136	0.7276	0.9323	0.049*	
N2	−0.0118 (6)	0.8522 (2)	0.8633 (2)	0.0428 (7)	
C1	0.6164 (11)	0.8151 (4)	0.5209 (4)	0.0677 (12)	
H1B	0.7036	0.7692	0.4657	0.081*	
C2	0.6554 (8)	0.9240 (3)	0.5124 (3)	0.0527 (10)	
C3	0.5303 (9)	0.9909 (3)	0.5956 (3)	0.0568 (9)	
H3A	0.5619	1.0647	0.5907	0.068*	
C4	0.3591 (9)	0.9509 (3)	0.6861 (3)	0.0544 (10)	
H4A	0.2762	0.9975	0.7417	0.065*	
C5	0.3098 (8)	0.8405 (3)	0.6946 (3)	0.0466 (8)	
C6	0.4448 (10)	0.7733 (3)	0.6129 (4)	0.0616 (11)	
H6A	0.4209	0.6992	0.6193	0.074*	
C7	−0.3375 (7)	0.8492 (3)	1.0262 (3)	0.0409 (7)	
C8	0.1202 (8)	0.7935 (3)	0.7861 (3)	0.0466 (8)	
H8A	0.0950	0.7192	0.7881	0.056*	
C9	−0.5355 (7)	0.7844 (3)	1.0984 (3)	0.0398 (7)	
C10	−0.6880 (9)	0.8080 (3)	1.1980 (3)	0.0483 (9)	
H10A	−0.6893	0.8733	1.2393	0.058*	
C11	−0.8474 (9)	0.7132 (3)	1.2281 (3)	0.0515 (9)	
H11A	−0.9722	0.7044	1.2928	0.062*	
C12	−0.7811 (9)	0.6394 (3)	1.1444 (3)	0.0513 (9)	
H12A	−0.8536	0.5694	1.1428	0.062*	
O1W	−0.1978 (8)	0.5707 (2)	0.8764 (3)	0.0596 (8)	
H1W	−0.037 (11)	0.526 (4)	0.906 (4)	0.084 (15)*	
H2W	−0.372 (13)	0.537 (4)	0.893 (4)	0.093 (18)*	

### Atomic displacement parameters (Å^2^)

**Table:**

	*U*^11^	*U*^22^	*U*^33^	*U*^12^	*U*^13^	*U*^23^
Cl1	0.0710 (7)	0.0849 (8)	0.0595 (5)	−0.0026 (6)	0.0271 (5)	0.0050 (5)
O1	0.0543 (16)	0.0340 (13)	0.0700 (15)	−0.0032 (11)	0.0163 (12)	−0.0029 (12)
O2	0.0529 (15)	0.0354 (12)	0.0497 (13)	−0.0044 (11)	0.0163 (11)	−0.0023 (10)
N1	0.0388 (16)	0.0333 (14)	0.0522 (15)	−0.0034 (12)	0.0113 (12)	0.0006 (13)
N2	0.0353 (15)	0.0413 (15)	0.0521 (16)	−0.0009 (13)	0.0071 (13)	0.0022 (13)
C1	0.081 (3)	0.064 (3)	0.060 (2)	0.003 (2)	0.034 (2)	−0.012 (2)
C2	0.044 (2)	0.064 (3)	0.051 (2)	−0.0025 (18)	0.0087 (16)	−0.0037 (17)
C3	0.065 (2)	0.050 (2)	0.056 (2)	−0.007 (2)	0.0161 (18)	−0.0020 (19)
C4	0.060 (2)	0.048 (2)	0.058 (2)	−0.0010 (18)	0.0225 (18)	−0.0051 (18)
C5	0.042 (2)	0.051 (2)	0.0475 (19)	−0.0007 (17)	0.0098 (15)	−0.0014 (16)
C6	0.072 (3)	0.051 (2)	0.063 (2)	−0.008 (2)	0.025 (2)	−0.0125 (19)
C7	0.0359 (18)	0.0370 (18)	0.0503 (18)	0.0050 (15)	0.0092 (14)	0.0040 (15)
C8	0.044 (2)	0.0413 (18)	0.0550 (19)	−0.0018 (16)	0.0117 (16)	−0.0042 (16)
C9	0.0392 (19)	0.0361 (17)	0.0442 (16)	0.0035 (15)	0.0045 (14)	0.0028 (14)
C10	0.052 (2)	0.045 (2)	0.0493 (18)	0.0023 (16)	0.0125 (16)	−0.0033 (16)
C11	0.054 (2)	0.048 (2)	0.053 (2)	0.0092 (17)	0.0221 (17)	0.0093 (17)
C12	0.059 (2)	0.042 (2)	0.055 (2)	−0.0053 (17)	0.0195 (18)	0.0072 (16)
O1W	0.053 (2)	0.0392 (14)	0.087 (2)	−0.0005 (14)	0.0169 (16)	−0.0001 (14)

### Geometric parameters (Å, °)

**Table:**

Cl1—C2	1.735 (4)	C4—H4A	0.9300
O1—C7	1.244 (4)	C5—C6	1.384 (5)
O2—C12	1.356 (4)	C5—C8	1.466 (5)
O2—C9	1.379 (4)	C6—H6A	0.9300
N1—C7	1.342 (4)	C7—C9	1.461 (5)
N1—N2	1.386 (4)	C8—H8A	0.9300
N1—H1A	0.8600	C9—C10	1.346 (5)
N2—C8	1.282 (4)	C10—C11	1.427 (6)
C1—C2	1.368 (6)	C10—H10A	0.9300
C1—C6	1.396 (6)	C11—C12	1.339 (5)
C1—H1B	0.9300	C11—H11A	0.9300
C2—C3	1.372 (5)	C12—H12A	0.9300
C3—C4	1.375 (5)	O1W—H1W	0.97 (5)
C3—H3A	0.9300	O1W—H2W	0.92 (6)
C4—C5	1.393 (5)		
			
C12—O2—C9	106.2 (3)	C5—C6—H6A	119.5
C7—N1—N2	119.7 (3)	C1—C6—H6A	119.5
C7—N1—H1A	120.1	O1—C7—N1	123.9 (3)
N2—N1—H1A	120.1	O1—C7—C9	120.2 (3)
C8—N2—N1	114.6 (3)	N1—C7—C9	115.8 (3)
C2—C1—C6	119.6 (4)	N2—C8—C5	121.7 (3)
C2—C1—H1B	120.2	N2—C8—H8A	119.2
C6—C1—H1B	120.2	C5—C8—H8A	119.2
C1—C2—C3	119.7 (4)	C10—C9—O2	109.7 (3)
C1—C2—Cl1	119.8 (3)	C10—C9—C7	131.6 (3)
C3—C2—Cl1	120.5 (3)	O2—C9—C7	118.6 (3)
C2—C3—C4	121.3 (4)	C9—C10—C11	106.7 (3)
C2—C3—H3A	119.4	C9—C10—H10A	126.6
C4—C3—H3A	119.4	C11—C10—H10A	126.6
C3—C4—C5	120.0 (4)	C12—C11—C10	106.2 (3)
C3—C4—H4A	120.0	C12—C11—H11A	126.9
C5—C4—H4A	120.0	C10—C11—H11A	126.9
C6—C5—C4	118.4 (4)	C11—C12—O2	111.1 (3)
C6—C5—C8	119.1 (3)	C11—C12—H12A	124.4
C4—C5—C8	122.5 (3)	O2—C12—H12A	124.4
C5—C6—C1	121.0 (4)	H1W—O1W—H2W	109 (5)
			
C7—N1—N2—C8	178.5 (3)	C6—C5—C8—N2	−179.5 (3)
C6—C1—C2—C3	1.2 (7)	C4—C5—C8—N2	0.2 (6)
C6—C1—C2—Cl1	−178.5 (3)	C12—O2—C9—C10	−1.6 (4)
C1—C2—C3—C4	−1.7 (6)	C12—O2—C9—C7	180.0 (3)
Cl1—C2—C3—C4	178.0 (3)	O1—C7—C9—C10	−6.7 (6)
C2—C3—C4—C5	0.0 (6)	N1—C7—C9—C10	172.3 (4)
C3—C4—C5—C6	2.1 (6)	O1—C7—C9—O2	171.4 (3)
C3—C4—C5—C8	−177.6 (3)	N1—C7—C9—O2	−9.6 (4)
C4—C5—C6—C1	−2.6 (6)	O2—C9—C10—C11	1.1 (4)
C8—C5—C6—C1	177.1 (4)	C7—C9—C10—C11	179.3 (3)
C2—C1—C6—C5	1.0 (7)	C9—C10—C11—C12	−0.3 (4)
N2—N1—C7—O1	−4.2 (5)	C10—C11—C12—O2	−0.7 (4)
N2—N1—C7—C9	176.9 (3)	C9—O2—C12—C11	1.4 (4)
N1—N2—C8—C5	177.2 (3)		

### Hydrogen-bond geometry (Å, °)

**Table:**

*D*—H···*A*	*D*—H	H···*A*	*D*···*A*	*D*—H···*A*
O1W—H1W···O1^i^	0.97 (5)	1.90 (5)	2.865 (4)	174 (5)
O1W—H2W···O1^ii^	0.92 (6)	1.97 (6)	2.873 (4)	168 (5)
N1—H1A···O2	0.86	2.36	2.715 (4)	106
N1—H1A···O1W	0.86	2.05	2.886 (4)	165
C8—H8A···O1W	0.93	2.49	3.290 (5)	144

Symmetry codes: (i) −*x*, *y*−1/2, −*z*+2; (ii) −*x*−1, *y*−1/2, −*z*+2.
